# Gaze aversion as a cognitive load management strategy in autism spectrum disorder and Williams syndrome

**DOI:** 10.1111/j.1469-7610.2011.02481.x

**Published:** 2011-10-26

**Authors:** Gwyneth Doherty-Sneddon, Deborah M Riby, Lisa Whittle

**Affiliations:** 1 School of Life Sciences, Northumbria UniversityNewcastle upon Tyne; 2 School of Psychology, Newcastle UniversityNewcastle upon Tyne; 3 School of Natural Sciences, Stirling UniversityStirling, UK

**Keywords:** Eye contact, gaze, Williams syndrome, gaze aversion, autism spectrum disorder

## Abstract

**Background:**

During face-to-face questioning, typically developing children and adults use gaze aversion (GA), away from their questioner, when thinking. GA increases with question difficulty and improves the accuracy of responses. This is the first study to investigate whether individuals with autism spectrum disorder (ASD; associated with reduced sociability and atypical face gaze) and Williams syndrome (WS; associated with hypersociability and atypical face gaze) use GA to manage cognitive load during face-to-face interactions.

**Methods:**

Two studies were conducted exploring the typicality of GA during face-to-face questioning in (a) ASD and (b) WS.

**Results:**

In Study 1, children with ASD increased their GA as question difficulty increased. In addition, they used most GA when thinking about their responses to questions, mirroring evidence from typically developing children. An important atypicality for participants with ASD was a significantly higher level of GA when listening to interlocutors. In Study 2, participants with WS showed typical patterns of GA in relation to question difficulty and across different points of the interaction.

**Conclusions:**

Two different neuro-developmental disorders, both characterized by significant problems with executive control of attention and atypicalities of social interactions, exhibited generally typical patterns of GA. All groups used most GA while thinking about questions, and increased their GA as questions got harder. In addition, children with ASD showed elevated levels of GA while listening to questions, but not while thinking about or making their responses, suggesting that they sometimes fail to see the relevance of attending to visual cues rather than actively avoiding them. Results have important implications for how professionals interpret GA in these populations and for social skills training.

## Introduction

For humans as well as many other animals the eyes are a very significant part of the face. Eye gaze serves many functions; ranging from social and emotional to intellectual. Furthermore, gaze behaviour plays an important role in many aspects of child development. Measures of gaze provide insights into typical and atypical social, emotional and cognitive development. For example, there are developmental changes in how infants respond to observed head and eye gaze shifts over the first 36 months of life (Moore & Corkum, 1998; Doherty, Anderson, & Howieson, 2009) linked to the maturation of socio-cognitive systems.

## Gaze aversion and cognitive load

Typically, we spontaneously and consistently look away from the face of an interlocutor during cognitively demanding activity by engaging in the overt behavioural response of ‘gaze aversion’ (GA; Doherty-Sneddon, Bruce, Bonner, Longbotham, & Doyle, 2002; Glenberg et al., 1998). While GA occurs very little when people are listening to another person speak (Doherty-Sneddon et al., 2002; Glenberg et al., 1998), it predominantly occurs while thinking and (albeit to a lesser extent) while speaking. So, the occurrence of GA potentially reflects the need to concentrate on drawing information from memory and/or engage in online cognitive processing, such as speech-planning or computation (Doherty-Sneddon et al., 2002; Glenberg et al., 1998). Conversely, given that under normal circumstances speech perception may be facilitated by the processing of visual information from a speakers face (McGurk & MacDonald, 1976), having access to relevant visual cues is most beneficial while listening to a speaker. In other words, we attend to visual cues when they are most useful to us, but when we need to concentrate on internal cognitive processing we ‘ignore’ them by averting our gaze away from the person with whom we are interacting – the ‘cognitive load hypothesis’ of GA. Consistent with this interpretation is the finding that GA also occurs in response to objects other than faces, including video-cameras (e.g. Ehrlichman, Weiner, & Baker, 1974). It appears then that people do not just avert their eyes from faces when they are thinking (see Ehrlichman, 1981), but also from any potentially distracting stimulus.

Ehrlichman, Mici, Sousa, and Zhu (2007) report increases in saccadic eye movement rate (EMR) during nonvisual cognition even when no visual distraction is available in the environment. They propose that these eye movements are related to the processes of both retrieving and maintaining information from/in memory, with the same neural circuitry involved in searching in long term memory as with searching for information in the visual environment. In contrast to the cognitive load hypothesis, in this account the eye movements are nonfunctional by-products of cognition.

## GA in typical development

Empirical work suggests that children start to use GA whilst thinking (and, to a lesser extent, speaking) from around 5 years of age (e.g. Doherty-Sneddon et al., 2002; Phelps et al., 2006). Indeed, it has been argued that a significant developmental surge in the use of GA behaviours during thought occurs between 5 and 6 years of age (Phelps et al., 2006); a behaviour which continues to develop (less markedly) throughout the next 2 years. So, by the time children have reached 8 years of age they use GA like adults to help them manage cognitive load (Doherty-Sneddon & Phelps, 2005; Doherty-Sneddon et al., 2002). In contrast, 5-year-old children have been shown to use GA to a much lesser extent (about half the proportion of thinking time as older children and adults), and also fail to consistently increase their use of GA in response to increasingly difficult questions although some evidence for this does occur (Doherty-Sneddon et al., 2002; Phelps et al., 2006). Furthermore, quantitative increases in the use of GA across these age groups, there are concomitant qualitative changes in the nature of GA shifts: whilst 5-year-olds used predominantly rapid multidirectional ‘flicking’ movements and some sustained left lateral eye movements, the 8-year-olds used predominantly sustained rightward eye movements while answering verbal reasoning and mental arithmetic questions.

## Neuro-developmental disorders and eye gaze

Williams syndrome (WS) and autism spectrum disorder (ASD) are neuro-developmental disorders associated with atypical patterns of gaze behaviour, atypicalities of social functioning and intellectual impairment. In this study, we provide novel analyses of GA by participants in these groups, contrasting GA while listening, thinking and speaking as well as under conditions of differing cognitive load. These measures provide new ways of addressing the cognitive and social phenotypes of the groups. In addition, they afford new insights into the implications of gaze behaviour for information processing during face-to-face interaction for these populations.

### Williams syndrome

Williams syndrome is a rare neuro-developmental disorder (estimated prevalence 1:20,000, Morris & Mervis, 2000; but see Strømme, Bjørnstad, & Ramstad, 2002) caused by the microdeletion of approximately 25 genes on chromosome 7 (7q11.23; Donnai & Karmiloff-Smith, 2000). This developmental disorder is associated with mild to moderate intellectual impairment (Searcy, Lincoln, Rose, et al., 2004) that occurs alongside unique cognitive and social-behavioural phenotypes. The social characteristics are very different from those associated with the autism spectrum (Brock, Einav, & Riby, 2007). Individuals with WS show outgoing social behaviours that have been referred to as ‘hypersocial’ (e.g. Jones et al., 2000; Frigerio et al., 2006), they may treat everyone as their friend irrespective of familiarity (Gosch & Pankau, 1997), and during social engagement they may use intense eye contact (Mervis et al., 2003).

The initial evidence that individuals with WS attended to people (and their faces) in a way that was different from those developing typically came from research with young children by Mervis et al. (2003). In that research, during an encounter with their geneticist, toddlers with WS showed atypically prolonged and intense gaze towards the geneticist’s face (Mervis et al., 2003). An interest in looking at faces is also evident at an older age. Adolescents and adults with the disorder tend to fixate on faces in social scenes and movies for significantly longer than typically developing individuals (Riby & Hancock, 2008, 2009a).

Modulating attention may be problematic for individuals with WS (Lincoln, Lai, & Jones, 2002; Cornish, Scerif, & Karmiloff-Smith, 2007) and may be entwined with problems shifting gaze towards and away from faces (Riby et al., 2011). Research has suggested that frontal lobe dysfunction may contribute to aspects of the WS social phenotype (Rhodes, Riby, Park, Fraser, & Campbell, 2010) and atypical gaze behaviours (Porter, Coltheart, & Langdon, 2007). It is further proposed that individuals with WS have problems that are specific to attention disengagement (rather than engagement) and that these problems are especially clear when disengaging from faces (Riby et al., 2011; Riby & Hancock, 2009).

### Autism spectrum disorders

Autism spectrum disorders cover a range of pervasive developmental impairments that have a particular effect upon the way an individual functions and interacts socially. Autism is characterized by severe impairment of social functioning, a lack of interest in social interactions, and abnormal eye contact (e.g. Frith, 1989). Indeed many of the classic descriptions of the disorder focus on a lack of interest in others and the atypical use of gaze (e.g. Lord et al., 2000). Willemsen-Swinkels, Buitelaar, Weijnen, and van Engeland (1998) looked the gaze and social behaviours of children with pervasive developmental disorder (PDD; 11 of 19 had autism) in parent–child interactions. They found that several aspects of gaze behaviour were very similar for the high functioning PDD and controls, for example, the overall amount of mutual gaze. However, the high functioning children with PDD did show atypicalities in the timing of gaze. For example, they were less likely to precede their declarative pointing with a gaze towards the parent than matched children with specific language delay or typically developing children.

It has been proposed that some of the core cognitive deficits seen in autism can be interpreted in terms of deviant cognitive processing; namely executive dysfunction (e.g. Russell, 1997). Part of the problem may be an inability to disengage from salient objects or inhibit responses that are inappropriate. Interestingly, individuals with WS may also have deficits related to executive functioning (Rhodes et al., 2010). Problems with the executive control of attention would predict atypicalities in the modulation of gaze and use of GA in face-to-face interactions in both these developmental disorders. In surprising data, this team report that increasing question difficulty did in fact increase GA in a group of participants with WS (Doherty-Sneddon, Riby, Calderwood, & Ainsworth, 2009), albeit baseline levels of GA were generally lower in this group than their typically developing controls. This suggests that participants respond to conditions of high cognitive demand by looking away more from a questioner’s face, and hence their attention is not ‘stuck’ on the face.

Other factors that may influence the occurrence of GA in response to cognitive difficulty are associated with atypicalities of gaze and face processing evident in ASD and WS. In ASD, there is evidence of diminished gaze fixations towards faces that may be due to hypoactivation of the brain areas related to face processing (Dalton et al., 2005). Dalton et al. (2005) note that activation of both the amygdala and fusiform gyrus regions were positively associated with time spent looking at the eye region of faces by individuals with an ASD. They suggest a heightened emotional response associated with gaze fixation in ASD that may be associated with active avoidance of face contact. In contrast, Senju and Johnson (2009a, b) propose that in autism atypical gaze is associated with a failure to respond to social cues, rather than an active avoidance of them.

A crucial point to make here is that the literature on atypicality in gaze behaviour in both WS and ASDs relates primarily to explaining atypical *perception* of gaze and its impact on behaviour. In contrast, the focus of the current study is how *cognitive activity* in both these populations may or may not modulate face contact (with implications for eye contact). While passively receiving or perceiving visually communicative gaze is reported as atypical in previous literature, the gaze behaviours of these populations while engaged in other nonvisual cognitive activities has not been investigated to date. In this study, we investigate whether children with an ASD or with WS adapt the amount they avert their gaze from faces while they engage in nonvisual cognitive tasks.

The timing of face gaze and GA within the interaction is crucial to its function in relation to appropriately timed access to visual communicative cues as well as optimizing processing demands in relation to other task demands. There is no existing literature that documents these patterns of gaze within face-to-face interaction in WS or ASD. This has important implications in relation to what *drives* GA in these populations. Particularly whether GA may under some circumstances (as in typically developing individuals) be an adaptive response to, or at least a reflection of, the cognitive load of face-to-face interactions. Many people on the autism spectrum report that faces are highly distracting (Attwood, 1998). We have shown in earlier studies that enforcing extended face contact in typically developing children can produce very significant cognitive interference effects (Doherty-Sneddon et al., 2000) this may be even more so with children with an ASD or with WS.

In Experiment 1, we investigate whether children with ASD show (a)typical patterns of GA over the listening, thinking and speaking phases of a face-to-face question–answer interaction. If atypical gaze in ASD is due to a failure to recognize the significance of visual facial cues (e.g. Senju & Johnson, 2009b) we would expect less face looking (increased GA) when children with ASD listen to questions. In contrast, if children with ASD are hyper-aroused by faces (e.g. Dalton et al., 2005) we would expect elevated levels of GA during all stages of the interaction. In Experiment 2, we investigate GA in face-to-face interactions in WS. The novel aspect taken is that in addition to looking at GA during thinking/problem-solving (as in Doherty-Sneddon et al., 2009) we look across the whole interaction: listening, thinking and speaking phases of questioning.

It is hypothesized that:

1. Individuals with WS and ASD will show atypicalities in their overall level of GA.
  - Individuals with ASD will show more GA than typically developing individuals (either because of a failure to see the significance of visual cues, particularly while listening, or hyper-arousal associated with visual cues cross all phases of the interaction).
  - Individuals with WS will show less GA than typical controls across all phases due to their tendency to use prolonged face fixations.
2. Participants with ASD and WS will not moderate GA levels in response to thinking time cognitive difficulty of questions due to poor executive control of attention.

## Experiment 1: GA in ASD

### Method

#### Participants

Twenty participants with ASD (19 males) ranged from 11 to 17 years, mean 13 years 2 months. Nine attended the special education unit of a mainstream secondary school and 11 attended schools for pupils with additional educational needs. All parents confirmed that their child had previously been diagnosed with an ASD by a clinician and provided informed and written consent for their child to participate. Teachers completed the Asperger Syndrome Diagnostic Scale (ASDS; Smith Myles, Jones-Bock, Simpson, 2000), scoring each individual on cognitive, maladaptive, language, social and sensorimotor behaviours (mean score 27; *SD* 8.73; no cut off is applied for this scale but the higher the score the higher the severity of autistic functioning, maximum possible 50). The ASDS is a standardized test designed to aid in the identification of individuals (aged 5–18 years) who show the characteristics of functioning at the high end of the autism spectrum. Internal consistency for the measure is reported to be high and the measure is deemed both reliable and valid (Smith Myles et al., 2000). For the research reported here, it is particularly relevant that scores on the ‘social subscale’ of the ASDS ranged from 4 to 12 (group mean 8.05; maximum possible 13, higher score indicating more abnormality). Although there was some individual variability, the majority of individuals in the ASD sample showed problems with social functioning; for example 17 individuals in the ASD sample were endorsed by their teachers as fulfilling the criteria of ‘avoids or limits eye contact’ and teachers of 16 participants in the sample endorsed the item for ‘has difficulty understanding social cues (e.g. turn-taking in conversation, politeness)’. Although this sample may be relatively high functioning on the autism spectrum (a necessity due to the nature of task demands) the subscale items emphasize that these individuals have a range of problems with social interactions. Finally, for this ASD sample, 16 participants had more severe problems (shown by higher scores) for items on the social subscale than for the items on the other subscales of the ASDS.

Each individual in the ASD group was matched to a typically developing child (9 males and 11 females, mean chronological age 9 years 6 months, ranging from 5 to 13 years) on the basis of verbal ability using raw scores on the British Picture Vocabulary Scale II (BPVS II, Dunn, Dunn, Whetton, & Burley, 1997). An independent *t*-test showed that there was no significant difference between groups on BPVS scores (*p* > .05) although the ASD group was significantly older than the TD group *t*(38) = 5.93, *p* < .01 (see Table 1). For typically developing participants, teachers completed the Strengths and Difficulties Questionnaire (SDQ; Goodman, 2001), scoring each individual on emotional symptoms, conduct, hyperactivity, peer relationships and prosocial behaviour. To comply with our inclusion criteria, all typically developing participants scored within the ‘normal’ range for the total difficulties score (scoring between 0 and 11). All participants had normal or corrected-to-normal vision.

**Table 1:** Participant demographic data for experiments 1 and 2 (standard deviation in parentheses)

	Experiment 1		Experiment 2	
	
	ASD	Typical matches	WS	Typical matches
Chronological age^a^				
Mean	13:08 (1:04)	9:11 (2:04)	21:08 (7:09)	8:10 (1:11)
Range	11:06–17:07	5:08–13:03	10:01–35:00	5:11–12:10
Verbal MA^b^				
Mean	10:08 (2:11)	10:08 (2:10)	9:03 (1:11)	9:03 (1:11)
Range	6:00–17:00	6:02–12:00	5:00–12:00	5:01–12:01
Nonverbal Score^c^				
Mean	30 (4.24)	28 (5.44)	17 (4.81)	27 (4.60)
Range	21–35	18–34	7–29	15–33
ASDS				
Mean	27 (9)	–	–	–
Range	15–45			
SDQ				
Mean	–	2 (3)	–	3 (3)
Range		0–11		0–10

ASD, autism spectrum disorder; WS, Williams syndrome; ASDS, Asperger Syndrome Diagnostic Scale; SDQ; Strengths and Difficulties Questionnaire.

a Expressed as years:months.

b As assessed by the British Picture Vocabulary Scale II (BPVS II).

c As assessed by the Raven’s Coloured Progressive Matrices task (RCPM).

Further information on the characteristics of the ASD group is provided by their performance on the Raven’s Coloured Progressive Matrices task (RCPM: Raven, Court, & Raven, 1990). The RCPM is a measure of fluid intelligence that is used widely for research purposes and has good psychometric values, requiring the participant to match visuo-spatial patterns (van den Heuvel & Smits, 1994). Participants are required to match visual patterns to a target template across 36 trials (max score 36). Individuals in the ASD group (mean raw score 30) showed a trend towards better performance on this task than the TD matched group [mean raw score 27; *t*(38) = 1.72, *p* = .094; see Table 1], which may be predicted by the age difference between groups.

Demographic data for participants in Experiments 1 and 2 are given in Table 1.

#### Materials and design

Participants were video recorded during a question and answer session. The session included 27 mental arithmetic questions (9 easy, 9 medium, 9 hard). The participant’s teacher tailored the mental arithmetic questions to fit within the ability of the pupil in order that 80%–100% of the easy questions, 30%–80% of the medium questions, and 10%–30% of the hard questions could be answered correctly. The experimenter and the participant sat across from each other at a table (approximately 1–1.5 m apart). A video recorder was set up behind the experimenter to monitor the eye gaze behaviour of the participant. GA was coded during ‘listening’, ‘thinking’ and ‘speaking’ time. Listening time was defined as the period of time during which the experimenter was asking the question. Thinking time was from when the experimenter finishing asking a question to when the participant began their answer. Speaking time was the period of time during which the participant spoke their response. These three phases of interaction reflected the natural progression of the question–answer interaction between experimenter and participant and were not explicitly distinguished during the questioning. Each of these phases was analysed as a percentage of time spent averting gaze during that phase. For example, total time spent averting gaze during the thinking period divided by the total time used for thinking (cf. Doherty-Sneddon et al., 2002). The video records were viewed and reviewed as necessary to determine the amount of time spent in GA. Interjudge reliability was calculated for a random sample of the GA measurements from the video recordings (the same coders coded the video records from Experiments 1 and 2 and hence the reliability measurement here is for both studies).This calculation included all of the listening, thinking, and speaking aversion scores for each of the question types for 10% of the children in the sample. In total, 646 episodes were coded by two judges. The judges agreed on 91% of these classifications. Furthermore, the coders’ scoring for the duration of GA correlated significantly, *r* (645) = .98, *p* < .001.

#### Procedure

All participants were tested in a quiet classroom at their school. Participants were told they would be asked mathematics (mental arithmetic) questions (easy, medium and hard) and they were given the following instructions: they could use their fingers to help count, take as much time as needed to answer the question, ask if they needed a question repeated and that they would not be given feedback as to whether the answer was correct/incorrect. (Mean response times in Experiments 1 and 2 were: ASD: 6862 ms; ASD controls: 6362 ms; WS: 6631 ms; WS controls: 7989 ms. The groups did not differ in terms of how long they took to respond to questions [ASD: *t*(19) = 0.52, *p* = .609; WS: *t*(17) = 1.16, *p* = .263]. The ASD group did not differ in terms of how often they requested a repetition of a question compared with their controls: ASD mean: 4.7/27 questions; typical controls: 3.25/27 questions [*t*(19) = 0.940, *p* = .359]. In Experiment 2, WS participants requested repetitions significantly less than their matched controls: WS mean: 1.06/27 questions; typical controls: 3.72/27 questions [*t*(17) = 4.03, *p* < .001]). Prior to commencement the experimenter ensured that the participant understood the instructions. Next the experimenter instructed the participant to look directly at their eyes for approximately 2 s to serve as calibration to aid in the analysis of the eye gaze behaviour during the question and answer session that followed. The mental arithmetic questions were asked in a randomized order across levels of difficulty. The experimenter looked at the participant at the beginning of each question and maintained eye contact for as long as the participant required to provide their answer.

### Results

#### Task performance

The percentage of correct responses to the mental arithmetic questions was recorded for each participant at each level of question difficulty. A two-way ANOVA with Group as a between participant variable (ASD; typical development) and Question Difficulty (easy; moderate; hard) as a within participant variable was carried out on accuracy scores (as percentage of total questions asked). There was a significant effect of Question Difficulty, *F*(2,76) = 190.37, *p* < .001, η_p_^2^ = .83 (mean easy = 98%, *SD* 7.80; mean moderate = 72%, *SD* 17.30; mean hard = 37%, *SD* 23.90). Post hoc *t*-tests showed that each level of difficulty was significantly different from each of the others [easy–moderate *t*(39) = 9.02, *p* < .001; easy–hard *t*(35) = 16.96, *p* < .001; moderate–hard *t*(35) = 12.43, *p* < .001]. Accuracy decreased as question difficulty increased. Performance was comparable across groups (*p* = .897). The interaction between variables was not significant (*p* = .637).

#### Gaze aversion

**Figure 1:**
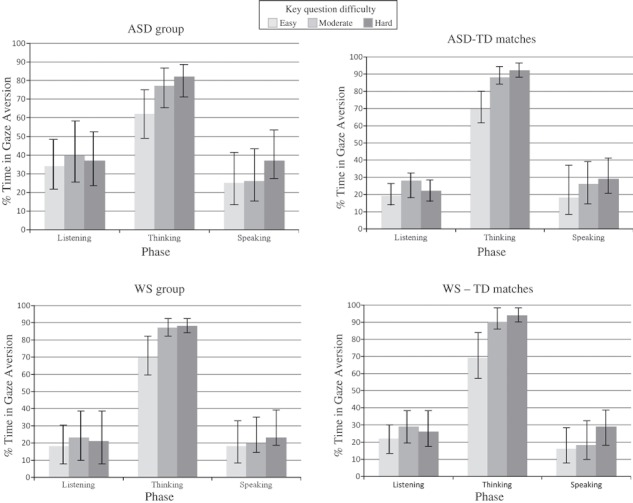
Proportion of time spent averting gaze across the three phases of the interaction and across the three levels of difficulty in Williams syndrome (WS); autism spectrum disorder (ASD) and typical development (error bars represent 95% confidence intervals)

The level of GA was significantly influenced by both the phase of the interaction (listening, thinking, speaking) and question difficulty (see Table 2 and Figure 1). Furthermore, while the total amount of GA by children with ASD was equivalent to the total amount used by the controls, children with ASD averted more while listening to the questions and less while thinking about the questions.

**Table 2:** Percentage of gaze aversion across interaction phase and level of task difficulty for participants with autism spectrum disorder (ASD) and typically developing matches (standard deviation in parentheses)

	Listening		Thinking		Speaking	
		
Difficulty	ASD	Typical matches	ASD	Typical matches	ASD	Typical matches
Easy	34 (33)	19 (13)	62 (33)	70 (18)	25 (32)	18 (28)
Moderate	40 (35)	28 (19)	77 (25)	88 (12)	26 (33)	26 (28)
Hard	37 (31)	22 (14)	82 (21)	92 (10)	37 (32)	29 (27)

A three-way mixed ANOVA was carried out on the GA data with factors Group (ASD; typical development), Question Difficulty (easy; moderate; hard), and Phase of Interaction (listening; thinking; speaking). The percentage of time participants spent averting their gaze was the dependent variable. Phase of interaction had a significant effect, with most GA when participants were thinking about their responses in contrast with listening or speaking, *F*(2,76) = 115.78, *p* < 001, η_p_^2^ = .75 (mean listening = 30%; thinking = 78%; speaking = 27%, see Table 2). Post hoc *t*-test showed that during the thinking phase GA was significantly greater than both listening and speaking phases [*t*(39) = 13.34, *p* < .001; *t*(39) = 12.26, *p* < .001, respectively]. There was no difference in GA between listening and speaking phases (*p* = .442).

Task Difficulty also had a significant effect on GA time, *F*(2, 76) = 23.26, *p* < .001, η_p_^2^ = .38 (mean GA during easy questions = 38%; moderate = 47%; hard = 50%; Mauchley’s test for sphericity showed that the homogeneity of variance assumption did not hold for this variable and so a Greenhouse–Geisser correction was applied). Post hoc *t*-tests showed that for easy questions participants used significantly less GA than when they were answering both moderately difficult questions and hard questions [*t*(39) = 4.58, *p* < .001; *t*(39) = 5.76, *p* < .001, respectively]. Participants also used less GA for moderately difficult questions compared with hard ones, *t*(39) = 2.08, *p* < .05.

There was a significant interaction between Group and Phase of Interaction, *F*(2,76) = 5.00, *p* < .01, η_p_^2^ = .12. Post hoc *t*-test showed that the ASD group used more GA while listening to questions than the typically developing controls, *t*(19) = 2.02, *p* < .05 (mean ASD = 37%; mean TD = 23%). In contrast, when thinking, typically developing children used more GA than the children with ASD, *t*(19) = 1.82, *p* < .05 (mean ASD = 74%; mean TD = 83%). There was no significant difference between groups for the speaking phase (*p* = .604).

Finally, there was a significant interaction between Phase of Interaction and Task Difficulty, *F*(4,152) = 7.73, *p* < .001, η_p_^2^ = .17 (Mauchley’s test for sphericity showed that the homogeneity of variance assumption did not hold for this variable and so a Greenhouse–Geisser correction was applied). Simple effects analysis showed that there was a significant effect of Task Difficult during thinking, speaking and listening [thinking: *F*(2,78) = 28.29, *p* < .001, η_p_^2^ = .42; speaking: *F*(2,78) = 6.68, *p* < .01, η_p_^2^ = .15; listening: *F*(2,78) = 5.98, *p* < .01, η_p_^2^ = .133]. The *F* values and effect sizes indicate that the effect of task difficulty was greatest during the thinking phase.

### Brief discussion

Children with ASD did not avert their gaze more than controls. However, the results showed that children with ASD averted their gaze considerably more while listening than typically developing participants. In contrast, while their level of GA peaked during the thinking phase (as for typically developing children) it actually remained at a lower level than the controls. So, when *thinking* about cognitively demanding information individuals who were developing typically averted the gaze more than those with ASD. Taken together these results suggest that children with ASD (even those who are relatively high functioning on the spectrum) fail to recognize the significance of visual social cues (cf. Senju & Johnson, 2009b) while listening to questions rather than actively avoid them as would be predicted by a theory of hyper-arousal or aversion to social stimulation (Dalton et al., 2005). It should be pointed out that while this is the case, even children in the ASD group looked at the experimenter 63% of the time in the listening phase (contrasting 26% of the time spent looking at the experimenter while thinking), again suggesting that face/eye contact is not actively avoided per se.

Participants in the ASD group avoided visual social cues during the thinking phase in a typical manner and increased their level of GA in response to an increase in cognitive demands. Previous research suggests wide-ranging deficits of executive functioning in this population (e.g. Russell, 1997) and therefore given the modulation of gaze in response to cognitive load that is reported here, the results suggests that increases of GA in response to cognitive load are not executively driven in this population.

## Experiment 2: GA in WS

### Method

#### Participants

Eighteen participants with WS (12 males) ranged from 10 to 35 years, mean 21 years 3 months. All individuals were recruited through existing links with the Williams syndrome Foundation. All participants had previously been clinically diagnosed and had previously had their diagnosis confirmed with positive fluorescent in situ hybridization (FISH) testing to detect the deletion of the *ELN* gene in the long arm of chromosome 7. All participants were reported to have normal or corrected-to-normal vision.

Each individual with WS was matched to a typically developing child (7 males, mean chronological age 8 years 4 months, ranging from 5 to 12 years) on the basis of verbal ability using raw score on the BPVS II (Dunn, Dunn, Whetton, & Burley, 1997). An independent *t*-test showed that there was no significant difference between groups (*p* > .05) for verbal ability although the WS group was chronologically older than the TD group [*t*(34) = 6.98, *p*<.001]. For typically developing participants, teachers completed the SDQ (Goodman, 2001), scoring each individual on emotional symptoms, conduct, hyperactivity, peer relationships and prosocial behaviour. To comply with our inclusion criteria, all typically developing participants scored with the ‘normal’ range for the total difficulties score (scoring between 0 and 11). All participants had normal or corrected-to-normal vision.

Further information on the WS sample comes from their performance on the RCPM (Raven et al., 1990). The RCPM has gained support for its use with individuals who have WS as a measure of nonverbal or spatial ability (e.g. van Herwegen, Farran, & Annaz, 2011). The participants with WS had raw scores between 7 and 29 (mean 17, see Table 1). These raw scores are comparable to those reported by van Herwegen et al. (2011) for individuals with WS. Individuals in the TD comparison group had RCPM scores between 15 and 33 (mean 27). The typical WS profile of deficits in visuo-spatial performance compared to verbal intelligence is supported by this cohort with WS; although the WS and TD did not differ statistically on verbal ability (BPVS scores, *p* > .05), individuals with WS performed significantly worse than the TD group on the RCPM *t*(34) = 6.23, *p* < .001).

All methods, stimuli and procedures replicate those used in Experiment 1. See Table 1 for participant demographics and refer to the previous footnotes.

### Results

#### Task performance

The percentage of correct responses to the mental arithmetic questions was recorded for each participant at each level of question difficulty. A two-way ANOVA with Group as a between participant variable (WS; typical development) and Question Difficulty (easy; moderate; hard) as a within participant variable was carried out on accuracy scores. There was a significant effect of Question Difficulty, *F*(2,68) = 292.85, *p* < .001, η_p_^2^ = .90 [mean easy = 95% (*SD* 8.40); mean moderate = 66% (*SD* 14.60); mean hard = 25% (*SD* 17.70)]. Post hoc *t*-tests showed that each level of difficulty was significantly different from each of the others [easy–moderate *t*(35) = 10.66, *p* < .001; easy–hard *t*(35) = 23.35, *p* < .001; moderate–hard *t*(35) = 13.89, *p* < .001]. Easy questions were answered more accurately than moderately difficulty questions which were answered more accurately than hard questions. Group had no effect on accuracy (*p* = .190) and the interaction between variables was not significant (*p* = .509).

#### Gaze aversion

The amount that participants averted their gaze away from the face of the experimenter was significantly influenced by both the phase of the interaction and question difficulty. Interestingly, participants with WS used similar amounts of GA to their matched controls and patterns of GA use across Phase and Task Difficulty were similar in both groups (see Table 3 and Figure 1). A three-way mixed design ANOVA was conducted using the GA data. Group was a between participant variable (WS; typical development) and Phase of Interaction (listening; thinking; speaking) and Question Difficulty (easy; moderate; hard questions) were within participant variables. Phase of interaction had a significant effect on the percentage of time spent averting gaze with most GA occurring while participants were thinking about their response *F*(2,68) = 168.80, *p* < .001, η_p_^2^ = .83 (mean listening = 23%; thinking = 83%; speaking = 21%). Post hoc *t*-tests showed that GA during thinking was significantly greater than during both listening and speaking phases [*t*(35) = 17.47, *p* < .001; *t*(35) = 17.00, *p* < .001, respectively]. There was no significant difference in GA length between the listening and speaking phases.

**Table 3:** Percentage of gaze aversion across interaction phase and level of task difficulty for participants with Williams syndrome and typically developing matches (standard deviation in parentheses)

	Listening		Thinking		Speaking	
			
Difficulty	WS	Typical matches	WS	Typical matches	WS	Typical matches
Easy	18 (26)	22 (22)	70 (30)	69 (30)	18 (26)	16 (26)
Moderate	23 (29)	29 (21)	87 (14)	90 (14)	20 (23)	18 (24)
Hard	21 (31)	26 (23)	88 (12)	94 (9)	23 (20)	29 (27)

Task Difficulty also had a significant effect on GA, *F*(2,68) = 16.51, *p* < .001, η_p_^2^ = .33 (mean GA during easy questions = 36%; moderate = 45%; hard = 47%). Post hoc *t*-tests showed that for easy questions participants used significantly less GA than when they were answering both moderately difficult questions and hard questions [*t*(35) = 4.28, *p* < .001; *t*(35) = 4.82, *p* < .001, respectively].

Finally, there was a significant interaction between Phase of Interaction and Task Difficulty. *F*(4,136) = 6.21, *p* < .05, η_p_^2^ = .15 (Mauchley’s test for sphericity showed that the homogeneity of variance assumption did not hold for this interaction and so a Greenhouse–Geisser correction was applied). Simple effects analysis showed that there was a significant effect of Task Difficulty at all phases of interaction, although effect sizes at each level differed considerably [listening: *F*(2,70) = 3.27, *p* < .05, η_p_^2^ = .09; thinking: *F*(2,70) = 20.69, *p* < .001, η_p_^2^ = .37; speaking: *F*(2,70) = 6.34, *p* < .05, η_p_^2^ = .15].

### Brief discussion

As in Experiment 1, hypothesis 1 was not supported by the WS data. Participants with WS averted their gaze to a similar degree as their typically developing counterparts and replicated the typical pattern, with more GA while thinking than listening or speaking.^1^ Furthermore, hypothesis 2 (that increasing question difficulty would not impact on GA in WS) was not supported. Participants with WS averted their gaze more as question difficulty increased.

This typical and consistent modulation of GA across the whole interaction is important as WS has previously been associated with a global tendency to over-gaze at interlocutors (e.g. Doyle et al., 2004). Here, we find no evidence of ‘over-gazing’ or ‘sticky’ fixation on faces and indeed entirely typical patterns of GA. One explanation for this may be that the current interactions involved participants engaging in problem-solving question–answer routines. This may well differ from more social forms of interaction that have been described in the previous literature on WS (Mervis et al., 2003) and is also very different to the type of face gaze explored using eye tracking tasks where participants are attending to an image on a screen that cannot make mutual eye contact (Riby & Hancock, 2008).

## General discussion

Neuro-developmental disorders such as WS and ASD are often associated with atypicalities of gaze behaviour. Important theoretical distinctions have been made suggesting a range of explanations for these atypicalities; from aversion to social stimuli (e.g. active avoidance in autism, Dalton et al., 2005) to a failure to learn the social rules or significance of social cues (passive avoidance in autism and over-gazing in WS, Senju & Johnson, 2009b; Mervis et al., 2003, respectively). The current experiments help us distinguish between these possibilities and offer a new cognitive load explanation of gaze behaviours.

This is the first study to differentiate different phases of interaction associated with listening, thinking and speaking in relation to gaze behaviour in ASD. We see that for this sample of individuals who are functioning on the autism spectrum, GA levels were primarily ‘typical’ especially in relation to the cognitive load of questions. Atypicality of gaze behaviour (for the parameters assessed here) was evident in the listening phase (more GA in ASD) and the thinking phase (less GA in ASD) of the interaction. It is likely that it is during the listening phase that an interlocutor (at that time a speaker) would notice reduced face gaze and hence why functioning on the autism spectrum is associated with reduced eye contact (Lord et al., 2000). In this study, children with autism averted their gaze more when listening, *less* while thinking and a similar amount while speaking compared to controls. The pattern of results allows us to draw an important speculative conclusion – that children with an ASD fail to recognize the significance of visual social cues while listening to questions rather than actively avoid them due to hyper-arousal or aversion to social stimulation. If their GA was driven by hyper-arousal or aversion to social stimuli we would expect elevated levels of GA across all phases of the interaction – not the pattern observed. Instead, we see elevated GA during listening, the point within interaction where typically developing children attend most closely to the face of their interlocutor when visual communication cues facilitate their understanding of questions (e.g. Doherty-Sneddon et al., 2002; Doherty-Sneddon & Kent, 1996).

We show here that there is in fact considerable GA during interactions with people with an ASD that looks, on the surface at least, relatively typical and indeed adaptive. Whether in fact the GA we see during thinking and speaking is functionally and qualitatively similar to that seen in typical development remains to be explored. In current on-going work, our team is using eye tracking technology to look in detail at the specific qualitative features of GA found in ASD, WS and typically developing children as saccadic activity has previously been reported to be different in these populations (Riby & Hancock, 2008). It will be interesting to see whether or not GA involves primarily sustained movements (previously associated with older children and adults in typical development, Doherty-Sneddon, Phelps, & Clark, 2007) or brief, rapid, saccadic movements (previously associated with earlier ontogenetic development, Doherty-Sneddon et al., 2007).

The current results are not consistent with previously reported hypersociability account of WS. The baseline levels and typical modulation of GA across the interaction by the participants with WS is important given that WS has previously been associated with a global tendency to over-gaze at interlocutors (Mervis et al., 2003). One explanation for the current findings may be that the problem-solving question–answer routines used here elicit very different patterns of gaze compared to more social encounters described in previous literature. In on-going work, we are directly comparing patterns of GA in problem-solving versus social types of interactions. In one of our own recent studies, participants with WS did show lower levels of GA compared with controls while engaged in moderate and difficult problem-solving (Doherty-Sneddon et al., 2009), although they still evidenced a significant increase in GA when thinking, especially about harder questions. What may have caused the lower baseline levels of GA in Doherty-Sneddon et al. (2009) which contrast with our current typical levels? In the 2009 study levels of thinking time, GA were generally lower than previously found even for the typical controls who averted their gaze only 56.9% of the time while thinking (cf. to 81.5% for typical controls in the current study; 77% of thinking time for typically developing 8-year-olds, Doherty-Sneddon et al., 2002). The GA paradigm we have used is generally very similar across studies. The main differences across studies are the experimenters who have questioned the children. The data from our Doherty-Sneddon et al. (2009) study suggest that the experimenter involved was particularly engaging for participants, with both typically developing and WS participants averting gaze less (i.e. looking more at her than we normally see). Explaining this is beyond the scope of this article, but the pattern of results across studies does suggest that there may well be a number of factors that will impact on the overall amount of face contact someone will engage in (including perhaps the communicative style of the experimenter). An important point is that in both studies participants with WS increased GA when thinking about answers to questions and increased GA as questions got harder. At least in some types of circumstances people with WS use entirely typical patterns and baseline levels of gaze and GA. So over-gazing is certainly not a given in WS.

Both ASD and WS are neuro-developmental disorders associated with significant deficits in executive functioning (Porter et al., 2007; Ozonoff et al., 1991; Rhodes et al., 2010). Here, we show that in both groups GA is associated with thinking. Furthermore, both populations increase GA as question difficulty increases (an entirely typical pattern). This suggests that our earlier model of mature GA behaviour as executively driven (Doherty-Sneddon et al., 2007) may not be accurate or may not apply to these concepts in atypical development. Based on our earlier work showing face-to-face interference effects when children are asked to look at faces while processing certain types of information (Doherty-Sneddon et al., 2000; Doherty-Sneddon, Bonner, & Bruce, 2001) and our finding that training children to increase their GA improves concentration and task performance (Phelps et al., 2006) we propose that the GA movements generated during challenging cognitive activity have a *functional* role to play in face-to-face interaction, even though they may be *automatically* generated.

The results may have important implications for informing practitioners and parents of individuals with WS or an ASD in relation to social skills training of eye contact. Our findings clearly show that while thinking, especially about difficult material, people need to/do look away from faces. Asking for eye contact during this phase of an interaction is only likely to interfere with concentration, working out a problem, or retrieving information from memory (Doherty-Sneddon et al., 2001). The atypically developing participants in the current studies had chronological and mental ages beyond 8 years of age. In typical development, 8-year-olds have adult-like patterns and rates of GA whereas younger children normally use less GA (Doherty-Sneddon et al., 2002). It may therefore be that younger children with an ASD or with WS may avert their gaze less than is evident here and extrapolation to younger children with an ASD or WS must therefore be made with caution. However, another important issue is whether GA functions in the same way to reduce cognitive load in younger rather than older participants (and indeed across the different populations documented here). In earlier work, we have shown that while 5-year-olds exhibit less GA than older children they nevertheless benefit from reducing face-to-face contact when concentrating on challenging material (Phelps et al., 2006), suggesting that GA does serve this function even in younger children. In ongoing work, we are investigating whether face-to-face interference effects occur for individuals functioning on the autism spectrum, and for those with WS, when participants are encouraged to engage in face contact while thinking. In other words, does looking too much at a face interfere with abilities to concentrate and answer questions correctly in these populations? The implications for social skills training are huge.

**Key points**

- WS and autism are associated with atypicalities of eye gaze.
- In typical development GA peaks when thinking and increases as cognitive difficulty of questions increases.
- Gaze aversion is indicative of thinking and cognitive challenge in WS and autism.
- Over-gazing in WS may be ‘situation dependent’.
- Reduced face gaze in autism is primarily associated with listening, possibly due to a lack of awareness of the value of visual cues.
- Social skills training must recognize the distinction between listening, thinking and speaking parts of an interaction in relation to gaze behaviour.
- Gaze aversion is often a sign of thinking in both typically and atypically developing populations. Social skills training must take this into account.
- Individuals with autism use relatively more GA while listening than typically developing counterparts. The impact of altering this with social skills training remains to be seen.

## Footnotes

1 In earlier work we have shown consistently that in typical development GA plateaus by 8 years of age (e.g. Phelps et al., 2006), therefore the lack of difference between WS group and their controls is not an artefact of the chronological age of the controls.

## Correspondence to

Gwyneth Doherty-Sneddon, School of Life Sciences, Northumbria University, Northumberland Building, Newcastle upon Tyne, NE1 8ST, UK; Tel: +44 (0) 191 227 4273; Fax: +44 (0) 191 227 4515; Email: gwyneth.doherty-sneddon@northumbria.ac.uk
